# The Influence of Mobile Device Type on Camera-Based Monitoring of Neck Movements for Cervical Rehabilitation

**DOI:** 10.3390/s23052482

**Published:** 2023-02-23

**Authors:** Maria Francesca Roig-Maimó, Iosune Salinas-Bueno, Ramon Mas-Sansó, Javier Varona, Pau Martínez-Bueso

**Affiliations:** 1 Department of Mathematics and Computer Science, University of the Balearic Islands, 07122 Palma, Spain; 2 Department of Nursing and Physiotherapy, University of the Balearic Islands and Health Research Institute of the Balearic Islands (IdISBa), 07122 Palma, Spain

**Keywords:** camera sensor, mobile device, neck, rehabilitation, serious game, mHealth

## Abstract

We developed a mobile application for cervical rehabilitation that uses a non-invasive camera-based head-tracker sensor for monitoring neck movements. The intended user population should be able to use the mobile application in their own mobile device, but mobile devices have different camera sensors and screen dimensions that could affect the user performance and neck movement monitoring. In this work, we studied the influence of mobile devices type on camera-based monitoring of neck movements for rehabilitation purposes. We conducted an experiment to test whether the characteristics of a mobile device affect neck movements when using the mobile application with the head-tracker. The experiment consisted of the use of our application, containing an exergame, in three mobile devices. We used wireless inertial sensors to measure the real-time neck movements performed while using the different devices. The results showed that the effect of device type on neck movements was not statistically significant. We included the sex factor in the analysis, but there was no statistically significant interaction between sex and device variables. Our mobile application proved to be device-agnostic. This will allow intended users to use the mHealth application regardless of the type of device. Thus, future work can continue with the clinical evaluation of the developed application to analyse the hypothesis that the use of the exergame will improve therapeutic adherence in cervical rehabilitation.

## 1. Introduction

Nowadays, mobile devices have become an indispensable tool for both daily work and leisure. Such devices provide, more and more, a wide variety of sensors and utilities that can be used to improve daily life. The camera sensor, the location and orientation devices and the sound capabilities are probably the most commonly used accessories.

Worldwide, mobile phones are used by 67% of the population from 16 to 64 years old, and by 96.6% of internet users, and their use is increasing by 1.8% per year. Other mobile devices, including tablet devices (34.8% of internet users from 16 to 64 years old, worldwide), smartwatches or smart wristbands (27.4% of internet users from 16 to 64 worldwide), are not so common, but their use is also increasing [1].

One of the areas where mobile device use has shown significant growth is health. Around 25% of internet users aged 16 to 64 worldwide use internet connection, with any device, for health-related purposes [1]. The use of mobile devices and applications for public health purposes (mHealth) is also increasing, with the number of mobile health applications growing exponentially.

Health applications are a valuable tool, as they can improve affordability and accessibility to geographically disperse or with low-income populations [2,3]. The use of mHealth has proven to be successful for management of several conditions management and follow-up (diabetes, mental condition or for heart-related conditions) [2,3,4,5,6], as well as for treatment adherence improvement. More recently, it has also been applied in other areas such as musculoskeletal rehabilitation.

Agnew et al. [3] and Ryan et al. [2] analysed mHealth apps for musculoskeletal conditions and evidence towards them, selecting those who fulfilled mHealth criteria of being user-centered apps (with or without clinical input), including exercise prescription, offering information on the developer, targeted body part and population, and of any existing evidence regarding their effectiveness. From the results, the conditions often addressed by mHealth were total knee or hip replacement or arthroplasty (14% and 7%), followed at a distance by other surgical procedures (anterior cruciate ligament reconstruction, shoulder joint replacement, subacromial decompression, with less than 7% each) and chronic conditions such as low back pain (14%), chronic knee pain or knee ostheoartritis (11%), neck pain (7%) and other less common conditions such as prolapsed intervertebral disk or tennis elbow (4% each).

Health applications for musculoskeletal rehabilitation are usually aimed towards education, pain management, pain therapy or self-management of musculoskeletal conditions, mainly by exercise prescription and compliance follow-up. They can have or not an input from the clinician. Although a clinician involved can be a limiting factor for the use of the health app, input from clinicians has proven to be effective in improving adherence [3].

Another potential way to increase adherence when using mHealth is by means of including an exergame, that is, a serious game that aims at physical exercise (in this case, therapeutic exercise) while entertaining the user. The use of this type of games would provide motivation, as well as monitoring and feedback, essential elements for adherence to treatment [7,8].

The common management of musculoskeletal conditions includes conservative treatments, which often include therapeutic exercise, with the aim of improving mobility, pain, function and quality of life [9,10,11]. Therapeutic exercise, usually supervised by a physiotherapist, includes mobility, strengthening, endurance and motor control exercises, always adapted to the treatment objectives, the body part involved and the musculoskeletal condition. The long-term beneficial effect is achieved by performing the therapeutic exercise constantly, so adherence to treatment becomes of utmost importance, especially when treatment is performed at home.

Neck pain is a multifactorial condition, and one of the most common musculoskeletal disorders, with a high prevalence and huge importance in terms of years lived with disability, as it can become chronic in 30–50% of cases [12,13]. Even though it is a frequent disease and of great importance in our society, neck pain has not been addressed much by mHealth applications. As seen before, few applications aimed at neck pain management can be found.

This could be due to the difficulty in the detection of cervical movement, and therefore, the possibility of providing feedback, or adapting to the movement capabilities or pain that the user presents at any time. In addition, as mentioned, feedback from the clinician can be a crucial element in the success of the treatment. Guidance, exercise selection and treatment under clinical criteria can also be essential elements for adherence and individualization of the treatment, and therefore, for its success.

With these objectives in mind, we developed a mobile application aimed at improving therapeutic exercise adherence in cervical rehabilitation. It uses a camera-driven head-tracker for mobile devices that we have previously developed to automatically detect and track the motion of the head, detecting and locating the nose of the user. The tracking method extracts facial features to precisely determine the position of the nose and to translate its movements into interaction actions to the device [14]. The method is based on facial feature tracking instead of tracking the overall head or face. The region of the nose has particular characteristics that make tracking easier: it is never occluded by facial hair or glasses and can always be seen by the camera when the user interacts with the device.

Then, the head-tracker was used as a sensor for the neck movements in the mobile application for cervical rehabilitation. The application was designed by clinical criteria and it consists of a first warm-up part, in which the user must perform some exercises (endurance, stretching, mobility) shown in videos. Afterwards, the user accesses the exergame. The designed exergame simulates a dart game, and user input combines the movement of the head, detected by the mobile head-tracker for the pointing action, and preset dwell-time for selection.

The configuration of target size, target location, order of targets, time of appearance and disappearance of the target, gain of the head-tracker and dwell-time criteria are set differently, depending on target size, target location, order of targets, time of appearance and disappearance of the target, according to preset user profiles. Finally, there is a phase of return to calm, also based on exercises shown in videos that the user must perform.

In a previous study [15], we proved that the camera-based head-tracker was feasible for rehabilitation purposes, in the matter of monitoring functional mobility, understood as the range of motion (ROM) used in daily life activities (estimated as 20% to 40% of maximum available neck ROM) [16,17]. We also proved the safety of the designed mobile application, checking that it is possible to use the head-tracker for neck movement detection in an exergame for cervical rehabilitation purposes and that it was safe in terms of the required neck ROM demanded by the exergame for some preset user profile.

When developing our health mobile application, one of our main goals is to achieve accessibility, i.e., that it can be used by as many people as possible, regardless of the mobile device they are using. However, the great variety of models, sizes and overall characteristics that can be found in the market of mobiles devices makes this a non-trivial task.

Therefore, in this paper, we study the influence of the type of mobile devices on the camera-based monitoring of neck movements for cervical rehabilitation purposes. In particular, we conduct an experiment to test if the above-mentioned characteristics of a mobile device affect the neck movements of the user when using the mobile application with the head-tracker.

## 2. Mobile Devices

We are interested in running our health application on mobile devices. The great variety of available models of devices also implies a great variety of screen sizes and resolutions which could affect the performance of a running application.

The impact of the screen size and resolution is a concern in the Human–Computer Interaction field and has been assessed in different terms such as usability and effectiveness. There has been interest in the search for an optimal device screen size [18], to investigate how the screen size and resolution can affect the user perception of image quality [19] or to analyse the effects of screen size and resolution on battery consumption [20]. However, as far as we know, no studies have been presented to assess how the size and resolution of the device can affect the performance and coherence of an input device.

**Figure 1:**
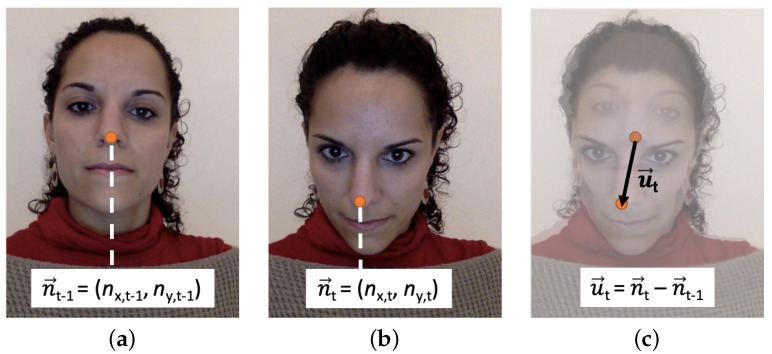
Calculation of ${\vec{u}}_{t}$ (**c**) from ${\vec{n}}_{t-1}$ (**a**) and ${\vec{n}}_{t}$ (**b**).

This affectation could be important in our case, as we aim to control the movement of the cursor on the screen using the movement of the head. Somehow, the tracking data from the head-tracker interface need to be translated to the device screen. The head-tracker returns the nose point of the user in the camera image at every time stamp. Figure 1a displays the nose point of the user, ${\vec{n}}_{t-1}$, returned by the head-tracker at the time stamp *t* − 1.

**Figure 2:**
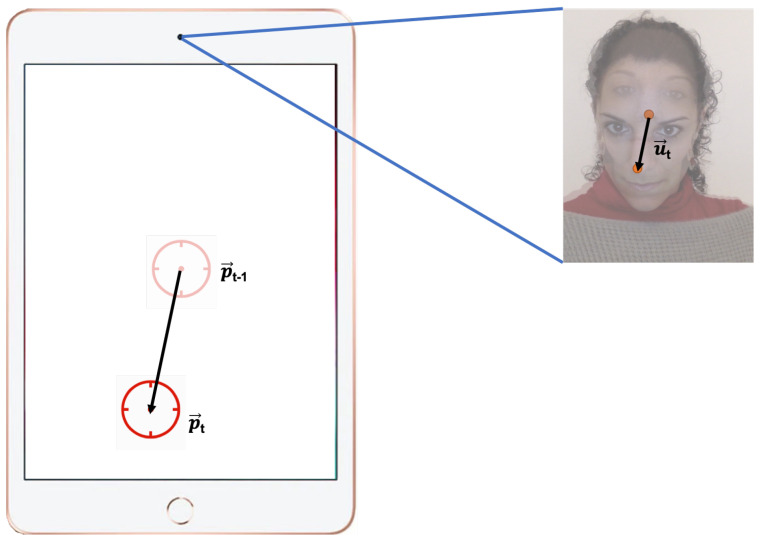
Translation of the change in coordinates of the nose of the user in the change in the coordinates of the cursor at the time stamp *t*.

We use a relative positioning approach, which means that each location of the cursor in the screen is calculated relative to the previous location rather than to a fixed location, using a transfer function. The transfer function translates the change in coordinates in the camera sensor ${\vec{u}}_{t}$ to a change in the coordinates on the device screen ${\vec{p}}_{t}$ (see Figure 2).

Let us define ${\vec{u}}_{t}$ as the user’s nose movement at every time stamp *t*, i.e., the change in coordinates of the nose point in the camera image:

$${\vec{u}}_{t}={\vec{n}}_{t}-{\vec{n}}_{t-1}$$

where ${\vec{n}}_{t}=\left(n_{x,t},n_{y,t}\right)$ corresponds to the nose point in the camera image at every time stamp *t* (see Figure 1).

The transfer function maps the user’s nose movement, ${\vec{u}}_{t}$, to a device screen position, ${\vec{p}}_{t}=\left(p_{x,t},p_{y,t}\right)$, at every time stamp *t*:

$${\vec{p}}_{t}={\vec{p}}_{t-1}+{\vec{u}}_{t}\cdot \vec{s}\cdot gainFactor,$$

where $\vec{s}=\left(s_{x},s_{y}\right)$ corresponds to a scale factor, and gainFactor is included to adjust the velocity of the input device, as explained later.

To ensure that users could reach all screen positions in a comfortable way with their range of head movement, a heuristic test was conducted to measure the users’ movement range in the camera image plane and compute the scale factor, $\vec{s}=\left(s_{x},s_{y}\right)$. For a source image of 192 × 144 pixels (low image resolution of the device’s camera), the informal test with five volunteers obtained a value of 55 pixels in both horizontal and vertical movement. Therefore, the scale factor was calculated as follows:

$$s_{x}=\frac{w}{55},s_{y}=\frac{h}{55},$$

where *w* and *h* correspond to the device display resolution (width, height).

We included a gain factor, that is, the amount of movement on the device in response to a unit amount of movement of the user in the camera image. The gain factor can be interpreted as the velocity of the cursor, in pixels/frame rate. For the developed health application, gain factor is one of the parameters that is crucial in terms of clinical criteria and rehabilitation goals. A high gain factor would allow users to perform despite having less mobility, while a low gain factor would imply a greater demand for movement, as well as greater motor control in those actions that require holding the cursor steady. Therefore, it is a parameter that should be carefully set depending on the user profile.

Previous work established that the head-tracker interface runs in real time (32 fps) and it is stable and robust for different users, light conditions, and a range of backgrounds [14]. Previous work also validated the interface from the point of view of Human–Computer Interaction (HCI) as a pointing device in target-selection tasks with able-bodied [21] and motor-impaired users [22]. A detailed technical description of our previously developed head-tracker interface can be found in Roig-Maimó et al. [14].

In this work, we aim to study the effect of the screen size and resolution of the devices in the neck ROM induced by the health application. Differences in these neck movements with distinct mobile devices would imply a recalculation of the gain factor, in order to assure that the mHealth application is device size-independent. We have to keep in mind that our goal is ensuring that our application can be used regardless of the mobile device used, making it accessible to as many people as possible.

## 3. Materials and Methods

The present experiment was conducted to test whether different characteristics of mobile devices affect the neck movements of the user when using a mobile application with a head-tracker.

### 3.1. Participants

Eighteen asymptomatic participants (ten females) were recruited from staff and students from a university campus in Spain. To be included, participants had to be aged 18 to 70. The average age was 43 years (*SD* = 11). They were excluded if they had reported or complained of neck, shoulder, and/or head impairments or if they had experienced pain in the preceding month.

### 3.2. Apparatus

**Figure 3:**
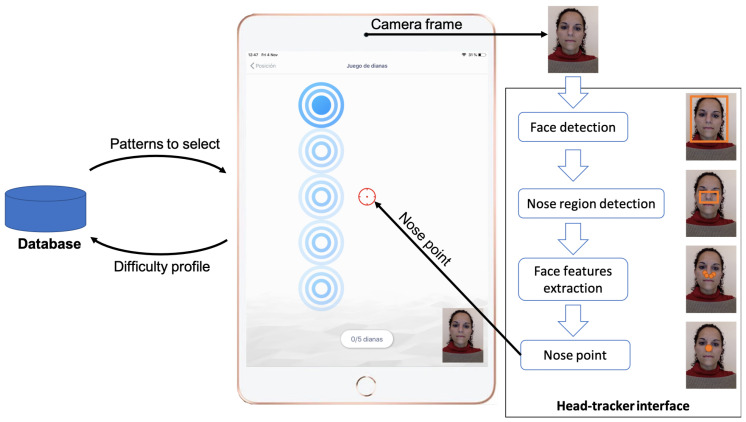
Architecture of the exergame. After selecting a difficulty profile, the mobile application sequentially presents a set of patterns of targets to select with the cursor (gun sight). The head-tracker interface captures a camera frame and returns the position of the nose of the user, which is translated in the position of the cursor through the transfer function described in Section 2.

For our experiment, we used our previously developed cervical rehabilitation exergame (see Figure 3) that simulates a dartboard game where the user has to select a series of targets of different sizes using the movement of the head as a pointing device (more detailed information of the exergame can be found in Salinas-Bueno et al. [15]).

In the application, we can select one of four different user profiles depending on the musculoskeletal disorders of the participants. This choice of profile determines, among others, the size, location and order of the targets, the speed of the required movements, whether time pressure is considered or not, gain factor and dwell-time criteria. In order to ensure that all the participants perform the same task for comparison reasons, we used exclusively a mid-difficulty default profile.

**Figure 4:**
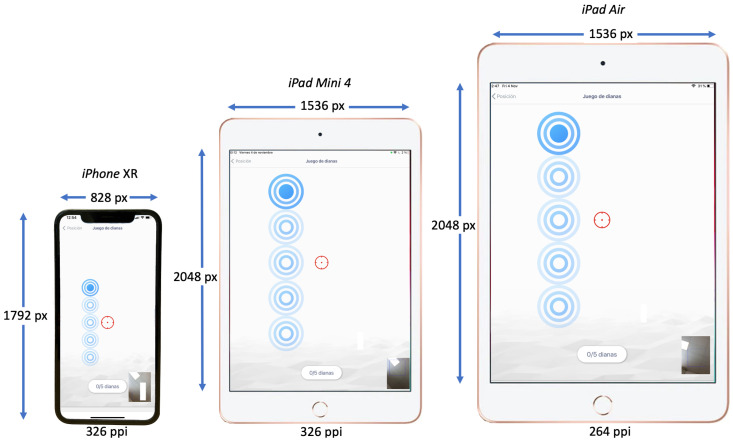
Screen sizes and resolutions of the Apple *iPad XR*, the Apple *iPad Mini 4* and the Apple *iPad Air*. As an illustration of the potential effect of the different screen sizes and resolutions of the devices, each of the devices displays the same view of the running application.

To conduct the experiment, we run the application on three different devices: An Apple *iPad XR*, an Apple *iPad Mini 4* and an Apple *iPad Air*, all of different sizes and resolutions. Figure 4 depicts the different screen sizes and resolutions of the used devices.

As seen in Figure 4, the biggest device, the Apple *iPad Air*, has a retina display of 9.7 inch (diagonal) with a resolution of 2048 × 1536 px and a pixel density of 264 pixels per inch (ppi). The middle-sized device, the Apple *iPad Mini 4*, has a retina display of 7.9 inch (diagonal) with a resolution of 2048 × 1536 px and a pixel density of 326 pixels per inch (ppi). The smallest device, the Apple *iPhone XR*, has a retina HD display of 6.1 inch (diagonal) with a resolution of 1792 × 828 px and a pixel density of 326 pixels per inch (ppi).

Figure 4 also shows a capture of the application running on each of the devices. It can be observed how the screen size and resolution of the device can influence the user-perceived distribution of the targets presented by the application.

**Figure 5:**
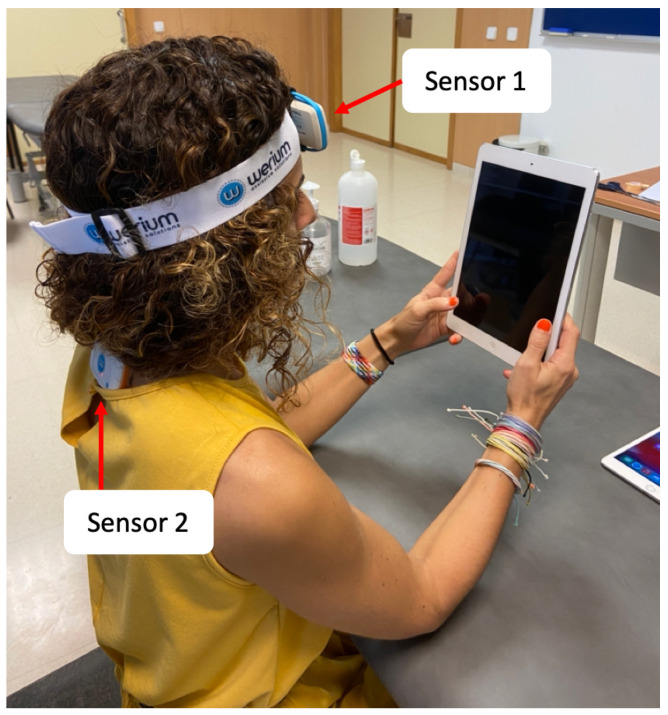
One of the participants ready to perform the experiment with the Apple *iPad Air*. ENLAZA inertial sensors placed on the forehead (see Sensor 1) and on T1–T2 thoracic vertebrae (see Sensor 2).

To measure the neck movements of the subjects while they were using the application, we used a software with two inertial sensors ENLAZA (Werium™system) [23] that measured and recorded the real-time movements performed by participants. The movements registered by these sensors and the software made the kinematic analysis possible. According to the protocol of the manufacturer, we placed one sensor on the forehead of the subject and another one on T1–T2 thoracic vertebrae (see *Sensor 1* and *Sensor 2* in Figure 5). With this location, Sensor 1 functioned as the movable arm of a goniometer and Sensor 2 functioned as the stationary part of the goniometer over the fulcrum of the movement, helping to measure real-time angle values in the coronal, sagittal and transverse anatomical planes.

#### 3.2.1. Procedure

The experiment was conducted following the guidelines of the Declaration of Helsinki, and it had previously been approved by the Research Ethics Committee of the University of the Balearic Islands (Exp.214CER21). All recorded data of the experiment were recorded in a file created expressly for this purpose and managed by the UIB according to personal data protection Regulation (EU) 2016/679. The experiment was led by one member of the research team and a collaborator, one of them a physiotherapist, with previous training on the procedure. The experiment consisted of performing one session playing the exergame, with the level of difficulty of the selected default profile, and using three different devices.

Explanation, information on the research in which they were going to participate and explanation of informed consent were given. Verification of compliance with the inclusion and exclusion criteria was also performed. Informed consent was therefore obtained from all subjects involved in the study. There was some previous data collection, such as sociodemographic data. Each subject was assigned a participant code that only one collaborator would know. The order of use of the devices was randomized.

The experiment was conducted in an experiment room. To prepare the room, we placed the measurement equipment as well as an adjustable stretcher and fix chair for the study subject.

Participants were seated on a fix chair in an upright position, with the stretcher height adjusted as a table, allowing to rest their elbows at a comfortable position to hold the mobile device naturally (see Figure 5). This position was also designed to control possible compensation movements (e.g., moving the mobile device instead of moving the head, or bending the whole body). Furthermore, participants were specifically instructed to avoid moving the device to interact with the application. The only requirement for holding the device was that the entire face had to be visible by the front camera of the device to start the head-tracker interface; this meant a minimum work distance of 15 cm, and at approximately 37 cm, a distance calculated to be comfortable for naturally holding the device. This is an intermediate reaching distance, and it was chosen after we checked that, within reaching range, there was no distance influence. Regarding the height of the camera, as the algorithm initialization required the full face of the user to be visible, the relative height between the camera and the line of sight of the user was constant regardless of the device used.

The researcher explained the exergame to the participant and asked them to select targets with the movements of their head. The researcher gave the participant the first device (randomly assigned) to start playing the exergame.

After the first device session was played, participants were asked to move freely their head and shoulders, in order to relax the muscles and joints involved. Then, they were delivered the second mobile device. The procedure was repeated with the three different devices. The complete experiment lasted about 15 min per participant.

The total number of sessions was 18 participants × 3 profiles = 54. During the sessions, the ROM of the participants was recorded in real time for each direction: flexion, extension, lateral flexion, and rotation.

#### 3.2.2. Design

The experiment was fully within-subjects with the *Device* as independent variable, which had the following levels: Apple *iPad Air*, Apple *iPad Mini 4* and Apple *iPhone XR*.

For each condition, the participants performed a session of the exergame (default profile) with the device corresponding to the level of the independent variable. The three device conditions were assigned applying a 3×3 Latin square.

A session of the default profile consists 32 patterns formed by 5 targets each. Therefore, in each session of the profile, the participant had to select 160 targets. The total number of targets to select by each participant was 160 targets × 3 devices = 480 targets. In total, the number of targets selected was 480 targets × 18 participants = 8640 targets (2880 targets per device).

The dependent variables were the maximum ROM achieved for all the directions of the movements of the cervical area: flexion, extension, right lateral flexion, left lateral flexion, right rotation and left rotation.

Following the Sex and Gender Equity in Research—SAGER—guidelines [24], we also investigated whether there was any difference related to sex over the dependent variables. For this reason, we gathered a balanced set of participants and we included the sex factor in the analysis. For the investigation of the effect of sex, we used a between-subjects design where the independent variable was Sex with the levels female and male.

## 4. Results

In this section, results are presented in terms of the maximum ROM achieved for each direction of the cervical area: flexion, extension, right lateral flexion, left lateral flexion, right rotation and left rotation. We first evaluate the effect of the within-subjects factor *Device* over the maximum degree achieved for each of the six movements and then we also investigate the effect of the factor sex over all the dependent variables.

### 4.1. Data Pre-Processing

For each of the participants, the raw data collected for each device condition consisted of the real-time neck mobility performed by the subject while using the application, reported by the ENLAZA inertial sensors (Werium™system [23]). Hence, for every participant, we gathered three files of raw data: one for each of the devices. Fitting a normal distribution to raw data, Shapiro–Wilk *W* tests indicate that the transformed data can be considered normal. Therefore, we can use *F*-Tests to find whether any differences exist among the device types.

In Table 1, there is a subset of the raw measures reported by the ENLAZA inertial sensors. It is formed by the degrees of movement at every time stamp for the anatomical planes: coronal, sagittal and transverse.

The translation between the measures of the anatomical planes and the directions of the cervical area is:

- Flexion: Negative values of the sagittal anatomical plane.
- Extension: Positive values of the sagittal anatomical plane.
- Right Lateral Flexion: Negative values of the coronal anatomical plane.
- Left Lateral Flexion: Positive values of the coronal anatomical plane.
- Right rotation: Positive values of the transverse anatomical plane.
- Left rotation: Negative values of the transverse anatomical plane.

For example, the value of −0.739 for the coronal anatomical plane corresponds to the right lateral flexion direction of movement and the value of 0.384 for the coronal anatomical plane corresponds to the left lateral flexion direction of movement.

To collect the maximum ROM achieved per participant for each direction of the cervical area, we selected the maximum of the values inside the 95% confidence interval (CI); considering as outliers the values above the 95th and below the 5th percentile.

### 4.2. Device Factor Analysis

**Figure 6:**
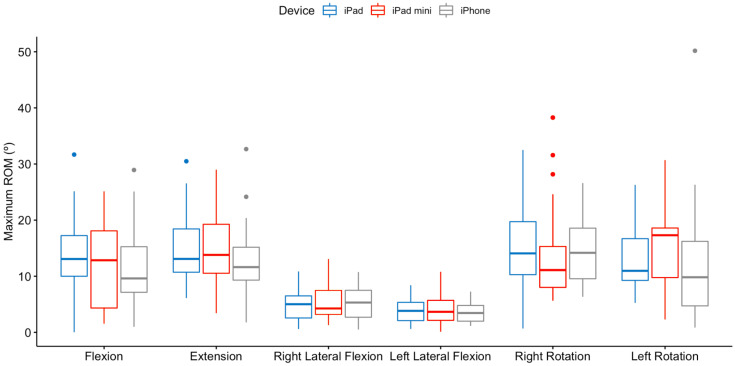
Maximum ROM achieved (in degrees) by device for each direction of the cervical area.

Figure 6 and Table 2 summarize the maximum ROM achieved by device for each direction of the cervical area. As seen in Table 2, in none of the directions of movement were the differences by device statistically significant.

### 4.3. Sex Factor Analysis

**Figure 7:**
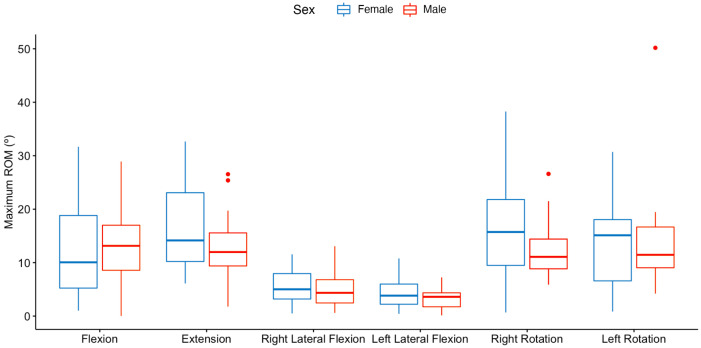
Maximum ROM achieved (in degrees) by sex for each direction of the cervical area.

Figure 7 and Table 3 summarize the maximum ROM achieved by sex for each direction of the cervical area.

As seen in Table 3, the only difference by sex that was statistically significant was for the right rotation direction of movement ($F_{1,52}=4.33$, p<0.05). The grand mean for the maximum degree achieved in right rotation movement was 14.8${}^{{}^{\circ}}$. By sex, the mean achieved by the female participants was 33.87% higher (16.6${}^{{}^{\circ}}$) than the mean achieved by males.

**Figure 8:**
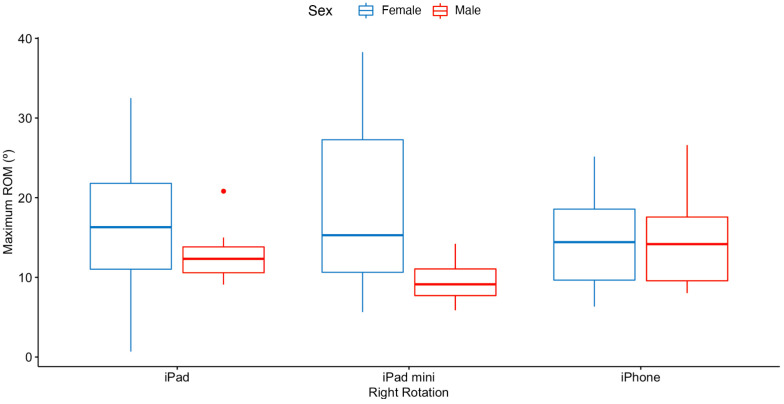
Maximum ROM achieved (in degrees) by sex for the right rotation movement for each device.

Figure 8 details the maximum ROM achieved for the right rotation movement by sex, where a significant difference was detected, for each of the three devices. If we analyze the interaction effect between the independent variables sex and device, a two-way ANOVA revealed that there was no statistically significant interaction between the effects of sex and device ($F_{2,32}=2.90$, p>0.05).

## 5. Discussion

In the previous section, we present the results of our experiment to investigate the effect of three mobile devices (*iPhone XR*, Apple *iPad Mini 4* and Apple *iPad Air*) in the maximum ROM achieved for all the directions of the cervical area while using our cervical rehabilitation exergame. The results show that there is no statistically significant difference in the maximum ROM achieved by our participants while using the application in any of the six directions of movement between the three mobile devices used for this experiment.

Having stated that the device type does not affect the maximum ROM required, we investigated whether there was a difference in the ROM achieved by sex in any of the directions of movement. The only difference that was statistically significant was for the right rotation direction of movement, were the females achieved a 33.87% higher rotation than the males. These results indicate that difference in rotation movement may be due to biomechanical reasons. In this regard, differences by sex in the biomechanical response of the neck have been studied [25,26,27,28]. According to the previous research, there are differences in neck muscle volume, global posture, posture of the cervical area, and in motion. These previous studies indicate that female spines show higher motion than males, in all directions of movement. Our findings confirm these results only for the right rotation movement. Therefore, further research would be necessary to confirm this difference.

Despite the detected difference in the right rotation movement, there was no statistically significant interaction between the effects of sex and device for the maximum ROM achieved in this direction of movement. Therefore, mobile device type is not a factor that influences neck performance in females or males.

We have not been able to contrast our results with those of other works. We have found attempts to find an optimal device screen size, to analyze the effect of size and resolution to image quality and consumption, but we have not found any studies to assess how the size and resolution of the device can affect the performance and coherence of a tracking device.

Our experiment was designed under the hypothesis that different mobile types (and their different screen size and resolution) would influence the movements detected by the application and therefore in the ROM asked to the users. If difference had been detected, that would have implied an adaptation of the gain factor value of the head-tracker transfer function to the different features of the mobile devices. As difference was not detected between devices, there is no need for adaptation or modification of the gain factor value.

**Figure 9:**
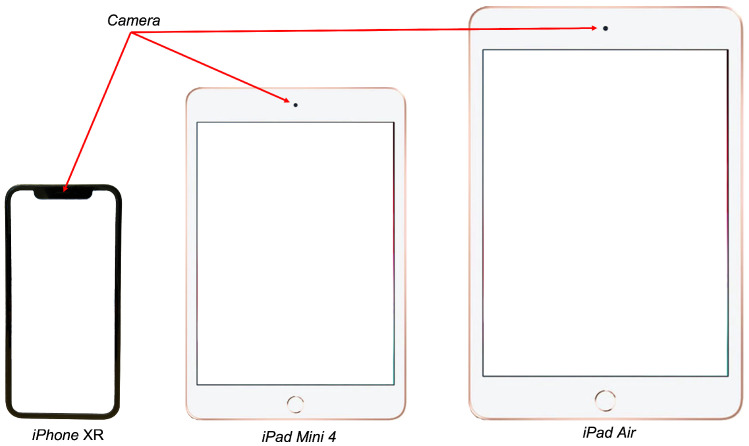
Camera location in the devices Apple *iPhone XR*, Apple *iPad Mini 4* and Apple *iPad Air*.

Nevertheless, all the devices used for the experiment have the camera placed in their upper center side (see Figure 9). As the placement of the camera affects the perspective of the captured image, this could alter the gain factor needed to assure the same ROM needed to perform the exercises of the exergame. Therefore, further analysis is needed, including mobile devices with different camera locations and different screen orientations of the application.

Once assured, as a result of this work, that the mobile application is device-agnostic, future work can continue with the clinical evaluation of the developed application to analyse the hypothesis that the use of the exergame will improve therapeutic adherence in cervical rehabilitation.

## Figures and Tables

**Table 1:** Example of the measures reported by the ENLAZA inertial sensors.

Time (ms)	Coronal	Sagittal	Transverse
2,301,952	−0.739	−1.721	2. 483
2,301,973	−0.178	−3	2.573
2,301,993	0.384	−4.12	2.663
2,302,014	0.384	−5.317	2.663

**Table 2:** Summary in terms of mean and standard deviation of the maximum ROM achieved by device for each direction of movement. Results of the *F*-Tests performed: value of the *F* Statistic and significance (* p<0.05).

Movement	iPad		iPad Mini		iPhone		*F*-Test	
	Mean	*SD*	Mean	*SD*	Mean	*SD*	$F_{2,34}$	sig.
Flexion	13.90${}^{{}^{\circ}}$	7.98${}^{{}^{\circ}}$	11.9${}^{{}^{\circ}}$	7.84${}^{{}^{\circ}}$	11.7${}^{{}^{\circ}}$	7.99${}^{{}^{\circ}}$	0.99	ns
Extension	15.60${}^{{}^{\circ}}$	6.84${}^{{}^{\circ}}$	15.10${}^{{}^{\circ}}$	7.76${}^{{}^{\circ}}$	13.20${}^{{}^{\circ}}$	7.09${}^{{}^{\circ}}$	1.19	p>0.05
Right Lateral Flexion	4.92${}^{{}^{\circ}}$	2.84${}^{{}^{\circ}}$	5.59${}^{{}^{\circ}}$	3.45${}^{{}^{\circ}}$	5.16${}^{{}^{\circ}}$	3.32${}^{{}^{\circ}}$	0.43	ns
Left Lateral Flexion	3.90${}^{{}^{\circ}}$	2.30${}^{{}^{\circ}}$	4.16${}^{{}^{\circ}}$	3.15${}^{{}^{\circ}}$	3.57${}^{{}^{\circ}}$	1.83${}^{{}^{\circ}}$	0.36	ns
Right rotation	14.80${}^{{}^{\circ}}$	7.22${}^{{}^{\circ}}$	14.80${}^{{}^{\circ}}$	9.55${}^{{}^{\circ}}$	14.70${}^{{}^{\circ}}$	6.01${}^{{}^{\circ}}$	0.003	ns
Left rotation	12.80${}^{{}^{\circ}}$	5.58${}^{{}^{\circ}}$	14.70${}^{{}^{\circ}}$	7.05${}^{{}^{\circ}}$	12.70${}^{{}^{\circ}}$	11.50${}^{{}^{\circ}}$	0.39	ns

**Table 3:** Summary in terms of mean and standard deviation of the maximum ROM achieved by sex for each direction of movement. Results of the *F*-Tests performed: value of the *F* Statistic and significance (* p<0.05).

Movement	Female		Male		*F*-Test	
	Mean	*SD*	Mean	*SD*	$F_{1,52}$	sig.
Flexion	12.4${}^{{}^{\circ}}$	8.44${}^{{}^{\circ}}$	12.6${}^{{}^{\circ}}$	7.22${}^{{}^{\circ}}$	0.003	ns
Extension	16.3${}^{{}^{\circ}}$	7.50${}^{{}^{\circ}}$	12.5${}^{{}^{\circ}}$	6.28${}^{{}^{\circ}}$	4	p>0.05
Right Lateral Flexion	5.51${}^{{}^{\circ}}$	3.2${}^{{}^{\circ}}$	4.86${}^{{}^{\circ}}$	3.16${}^{{}^{\circ}}$	0.552	ns
Left Lateral Flexion	4.42${}^{{}^{\circ}}$	2.76${}^{{}^{\circ}}$	3.20${}^{{}^{\circ}}$	1.85${}^{{}^{\circ}}$	3.441	p>0.05
Right rotation	16.6${}^{{}^{\circ}}$	8.8${}^{{}^{\circ}}$	12.4${}^{{}^{\circ}}$	4.98${}^{{}^{\circ}}$	4.33	* p<0.05
Left rotation	13.2${}^{{}^{\circ}}$	7.90${}^{{}^{\circ}}$	13.7${}^{{}^{\circ}}$	9.03${}^{{}^{\circ}}$	0.05	ns

## Data Availability

The data presented in this study are registered in the University of the Balearic Islands repository (data processing record code RAT034) and are available on request from the corresponding author.
